# A Hierarchical Bayesian Model for Crowd Emotions

**DOI:** 10.3389/fncom.2016.00063

**Published:** 2016-07-08

**Authors:** Oscar J. Urizar, Mirza S. Baig, Emilia I. Barakova, Carlo S. Regazzoni, Lucio Marcenaro, Matthias Rauterberg

**Affiliations:** ^1^Department of Industrial Design, Eindhoven University of TechnologyEindhoven, Netherlands; ^2^Department of Naval, Electric, Electronic, and Telecommunications Engineering, University of GenovaGenoa, Italy

**Keywords:** crowd behavior, emotion estimation in crowds, estimation of individual and collective emotions

## Abstract

Estimation of emotions is an essential aspect in developing intelligent systems intended for crowded environments. However, emotion estimation in crowds remains a challenging problem due to the complexity in which human emotions are manifested and the capability of a system to perceive them in such conditions. This paper proposes a hierarchical Bayesian model to learn in unsupervised manner the behavior of individuals and of the crowd as a single entity, and explore the relation between behavior and emotions to infer emotional states. Information about the motion patterns of individuals are described using a self-organizing map, and a hierarchical Bayesian network builds probabilistic models to identify behaviors and infer the emotional state of individuals and the crowd. This model is trained and tested using data produced from simulated scenarios that resemble real-life environments. The conducted experiments tested the efficiency of our method to learn, detect and associate behaviors with emotional states yielding accuracy levels of 74% for individuals and 81% for the crowd, similar in performance with existing methods for pedestrian behavior detection but with novel concepts regarding the analysis of crowds.

## 1. Introduction

The tendency of the urban development toward Smart cities (Chourabi et al., 2012) poses a number of research and engineering challenges among which crowd emotion management and prevention of escalations is of vital importance. Furthermore, with the fast growth of population in urban areas around the world, the phenomenon of crowds is set to become commonplace in the near future. The purpose of this work is to present an approach for estimating emotions of single individuals and of a crowd as a whole. In this work, we use the working definition of the crowd as a group of people in proximity, where a common motivation or set of emotions may exist as in the case of sports events and concerts, or merely individuals with different motivations and emotions walking around a busy area. In both instances, the term crowd behavior refers to the behavior adopted by individuals when becoming part of a crowd.

Research on crowd emotions differs significantly from the research on individual emotions in several ways. de Gelder (2006) and Huis in 't Veld and De Gelder (2015) propose that crowd emotions are a delicate balance between the emotions of the individuals and the emotions of the crowd. They found that the interactive or panicked crowds, as opposed to the individually fearful crowds, triggered more anticipatory and preparation action activity, whereas the brain was less sensitive to the dynamics of individuals in a happy or neutral crowd.

Despite the dissimilarities among the most prominent theories addressing crowds from psychologists and sociologist such as Le Bon (2001), Freud (1921), and Allett (1996) among others, there is a consensus on the important role of emotions in the phenomenon of crowd behavior (Challenger et al., 2009). Emotions can be thought as manifestations of our internal state of well-being utilizing psychophysiological and behavioral reactions. In more practical terms, emotions serve as a response to internal or external events experienced by a subject and are manifested over brief periods of time (Plutchik, 2011). Also, supported by the work done by Matsumoto (2004), Ekman et al. (1987) and Keltner and Ekman (2000) we have learn that emotions are discrete and measurable, also that certain emotions appear to be universally recognized despite cultural context or learned associations.

The use of facial and vocal expressions to infer emotions becomes unfeasible in crowded environments. Hence, in this work we propose the use of behaviors described as motion patterns to identify emotional states. This approach is supported by de Gelder et al. (2010), Van den Stock (2007) and Frijda (2010) where they suggest that the whole body expressions of emotion are primary carriers of emotion and action information and may thus play a more important role in a crowd situation than facial expressions. Emotions expressed by dancers and music instrument players have been simulated on robot agents by Barakova and Lourens (2010). The relationship between emotional states and behaviors is further supported by Damasio's Somatic Marker hypothesis (Bechara and Damasio, 2005) in which he explains that decision making involves both cognitive and emotional processes. However, it is important to point out that the relationship between emotions and behaviors is not straightforward as shown in appraisal theory (Moors et al., 2013) proposed by Arnold and developed Lazarus to explain how the same event can provoke different emotions in different individuals and occasions. Moreover, the contemporary appraisal theories define emotions as processes rather than states (Moors et al., 2013), which is in line with the modeling of the movement behavior of the individuals in the crowd.

The work presented in this paper proposes a hierarchical Bayesian model suited for crowded environments. Our model is capable to learn behaviors and associate them with emotions during the training phase, and to estimate emotional states based on partial observations of behaviors during the testing phase; we apply this for both the individuals in the crowd and the crowd as a whole. We consider two types of entities, namely the *individual* and the *crowd*. The entity *individual* describes the behavior and associated emotion of individual people walking across the observed environment. Hence, an instance of entity *individual* is implemented for each pedestrian detected. The entity *crowd* describes the whole congregation of people as a single being subjected to the laws of mental unity as explained in Le Bon (2001), hence having its own behavior and associated emotional state. In this work, we limit to describe emotional states in one dimension, ranging from positive to negative according to the principle of valence (Rosenhan and Messick, 1966).

**Figure 1:**
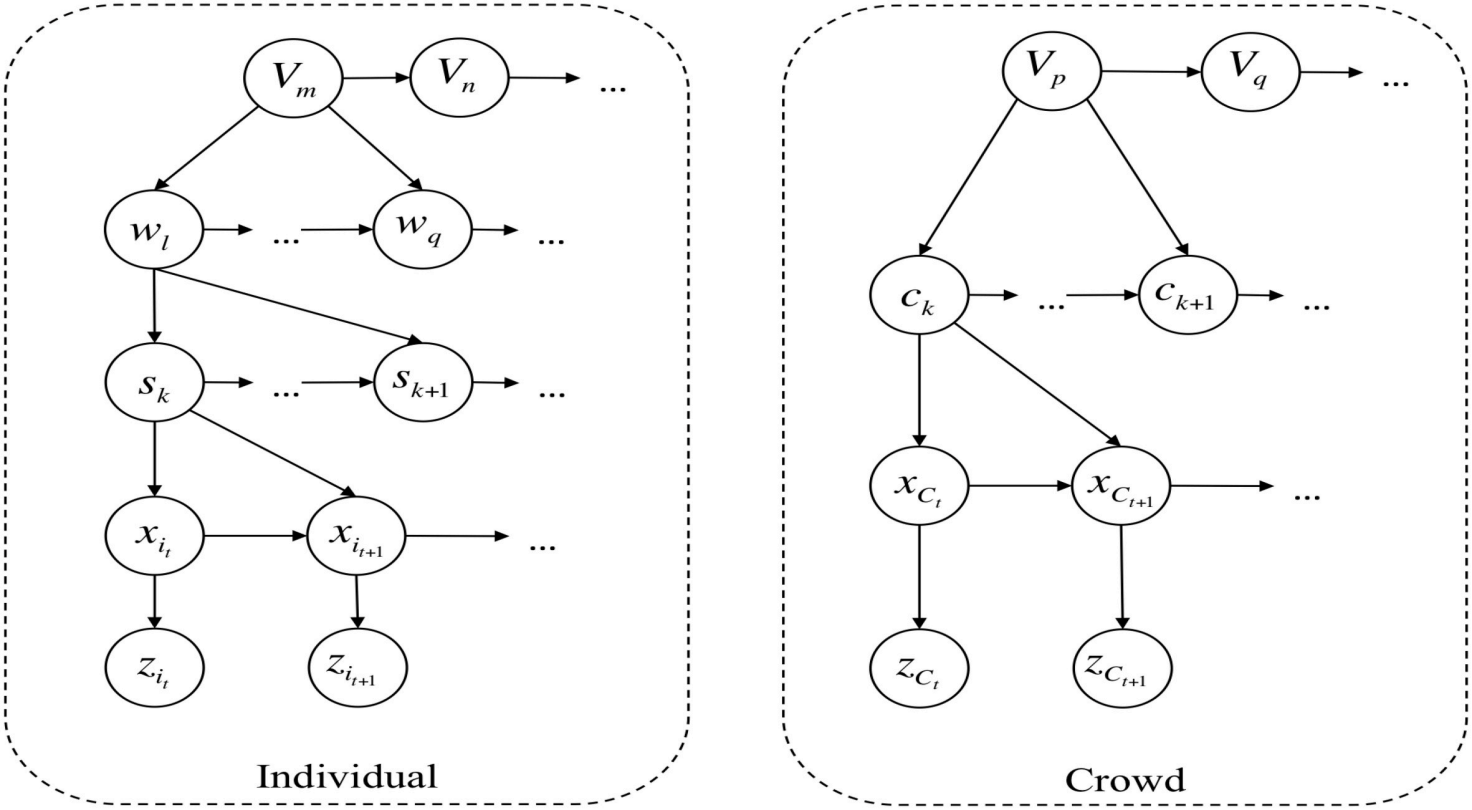
**Hierarchical bayesian model for entities ***individual*** and ***crowd*****.

In the proposed approach, the topology of an environment is learned from observed trajectories of individuals employing a self-organizing map *SOM*_*I*_, where each node of *SOM*_*I*_ represents a mutually exclusive zone. The path of individuals is expressed as a sequence of transitions among these zones, and a hierarchical Bayesian network builds probabilistic models to describe and group similar behaviors. The learned behaviors are associated with certain emotional states in an empirical fashion. We describe the configuration of the crowd in a given instant with a state vector containing the estimated density level in each zone of the environment. Employing a second self-organizing map *SOM*_*C*_ we cluster similar configurations of the crowd into a node of *SOM*_*C*_, enabling us to describe the behavior of a crowd as a transition of nodes of *SOM*_*C*_ using a similar hierarchical Bayesian network. The proposed hierarchical Bayesian model is presented in Figure 1. Finally, the emotional states are associated to crowd behaviors in an empirical way. The association between behaviors and emotions is done empirically because the interpretation of a behavior greatly depends on the context of the environment, for example, a fast-paced walk with sudden turns during rush-hour in a train station could have a different emotion associated if the same behavior was displayed in a museum.

The remaining of this work is organized as follow: Section 2 presents a brief survey of previous approaches to estimate emotions. A comprehensive description of our proposed model is presented in Section 3. Experiments and results to validate our model are given in Section 4. Finally, in Section 5 we state our conclusions and intended future work.

## 2. Related work

Most of the existing literature in the subject of human emotion recognition has been focused on individuals rather than crowds (Horlings et al., 2008; Izar, 2013). Facial and vocal expressions are useful indicators to infer emotional states, Ekman proposed in Ekman and Friesen (2002) a system based on sets of action units (AU) to recognize emotions based on facial movements. Juslin and Scherer explore the use of pitch and context to infer emotions, as presented in Juslin and Scherer (2008). However, the use of facial and vocal expressions to identify emotions is not feasible in crowded environments. Observation of behaviors seems more appropriate for estimating emotions in crowded scenarios but the relationship between behaviors and emotions is not straightforward as shown in Moors et al. (2013) as it varies depending on the context of the situation and environment. However, promising research has emerged in recent years proposing solutions to this problem. An interesting experiment in Novelli et al. (2013) tested a self-categorization theory to estimate positive and negative emotional responses to crowded environments under different circumstances. Inspired from the highly crowded cities in China, Liu et al. (2013) analyze the contagion of emotions among individuals, particularly under abnormal (panic) scenarios. A more relevant research and the starting point for the work presented here was done by Baig et al. (2014) with a probabilistic model to estimate emotional state of individuals as positive or negative based on the time and trajectory taken to traverse a simple scenario. Our contribution differs from Novelli et al. (2013) and Liu et al. (2013) in that we provide a method for online estimation of emotions of both individuals and the crowd as a whole. Also, unlike the work in Baig et al. (2014) that models only individuals that have the same motivation, we provide a more robust and adaptable framework that treats individuals and the crowd as separate entities to estimate emotions under environments where multiple types of behaviors are observed.

## 3. Methods

This section describes the proposed hierarchical Bayesian model to describe behaviors and associated emotional states of both entities *individual* and *crowd*. In this work, we define a behavior as the way in which an entity transits among different states to achieve its goal (destination). For *individual*, a state corresponds to a physical region of the environment whereas for the crowd a state corresponds to a given configuration of people's density distribution in the observed environment. Behaviors for both *individual* and *crowd* are labeled empirically by a human operator knowledgeable of the environment using the labels of positive, normal or negative to denote the emotional state.

In overall, our approach starts by learning the topology of the observed environment from the trajectory of individuals using a self-organizing map (SOM) (Kohonen, 1990) which divides the physical space into regions. Trajectories of individuals are represented as transition of regions, and all trajectories with similar destination are classified to a same behavior, to finally build a probabilistic model that describe this behavior. Likewise for the crowd, similar sequences of state transitions are grouped into a same behavior which is described by means of a probabilistic model. The bayesian network for both *individual* and *crowd* entities is presented in Figure 1.

Once the topology of the environment and behaviors of both the *individual* and the *crowd* are learned, we can test the ability of the model to produce estimation of emotions.

### 3.1. Environment representation

**Figure 2:**
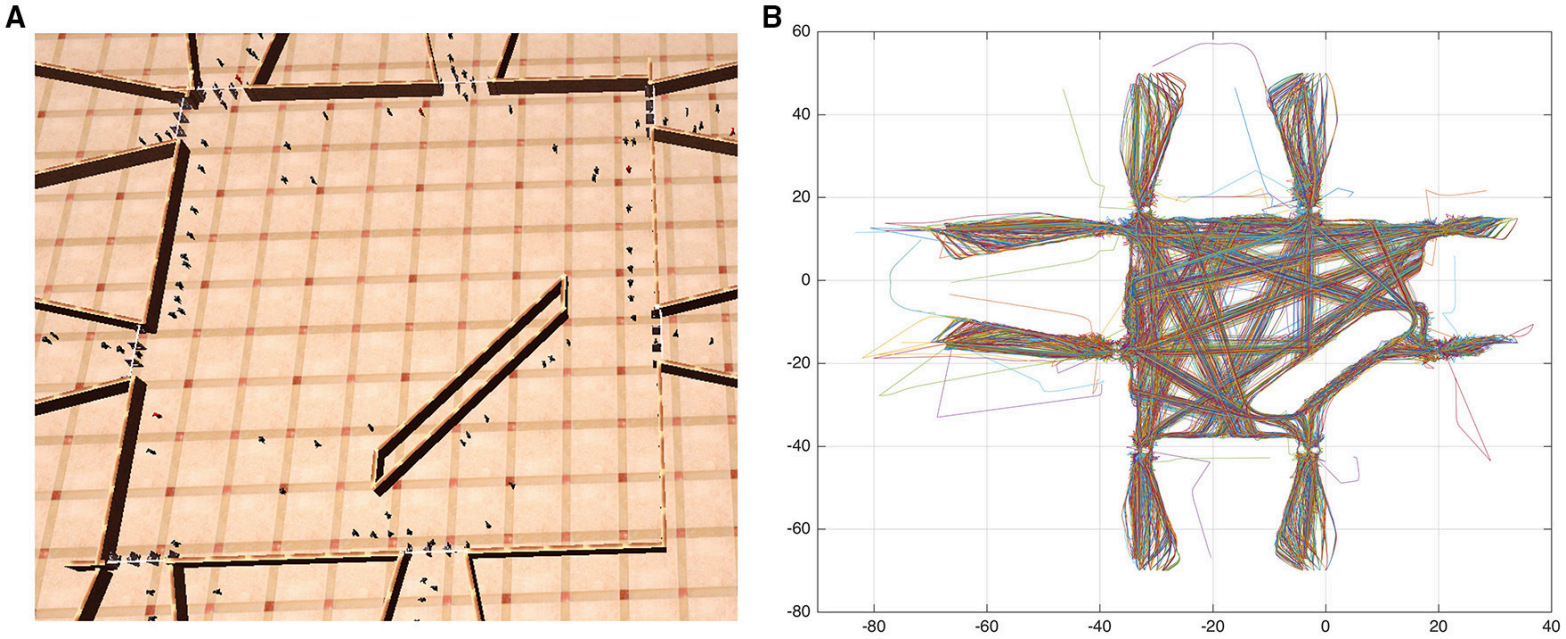
**(A)** Simulation of a crowded environment. **(B)** Plot of individual's trajectories, colors are assigned randomly.

**Figure 3:**
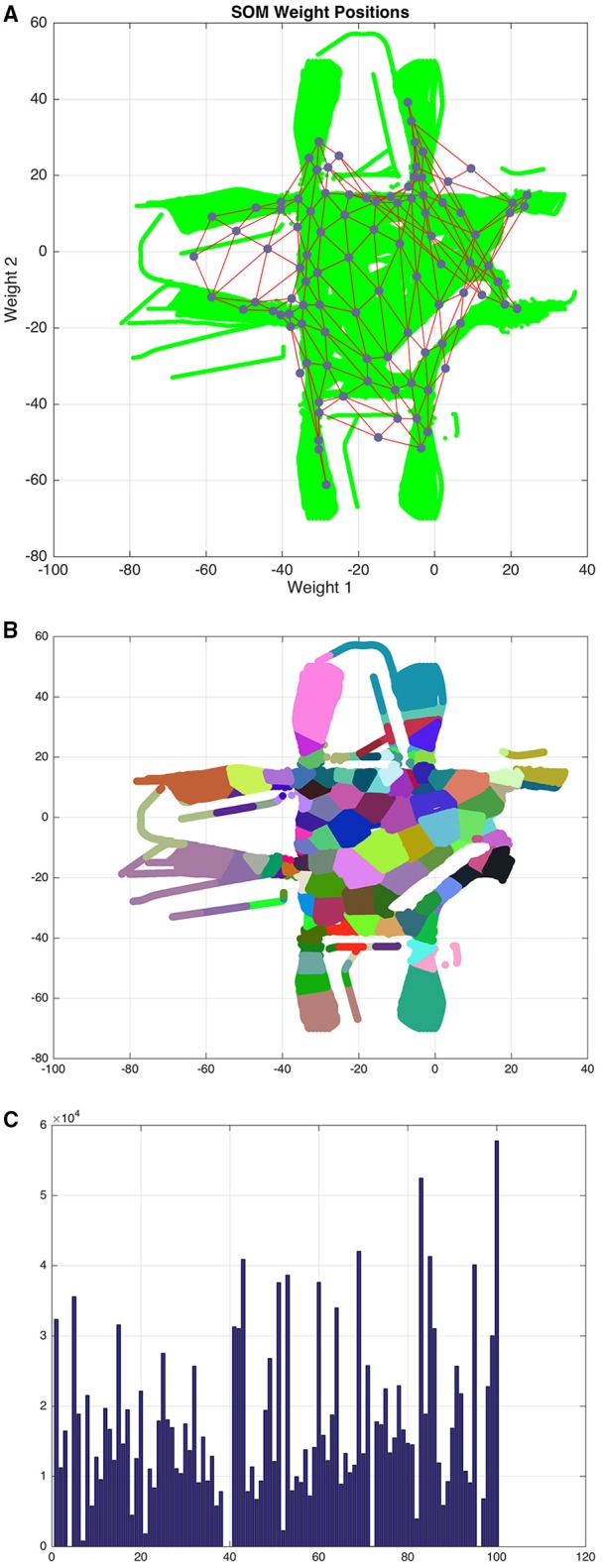
**(A)** Training data (green) and the self-organizing map *SOM*_*I*_ (red edges and blue nodes). **(B)** Environment partitioned into zones, colors are assigned to zones in a random fashion. **(C)** Clustering distribution of training data among zones.

We first address the problem of obtaining a topological representation of the environment of interest as this is necessary to describe behaviors of individuals. Let us consider an environment monitored by a surveillance camera that captures the motion of individuals as illustrated in Figure 2A. By applying state of the art techniques for multi-target tracking in camera networks (Antonini et al., 2006; Ali and Shah, 2008) it is possible to obtain the trajectory of each individual and collect this data into a training set **X**

(1) $$\begin{array}{l} \text{X}={\left\{{\hat{x}}_{1_{t}},\ldots ,{\hat{x}}_{N_{t}}\right\}}_{t=1}^{\tau_{k}} \end{array}$$

where ${\hat{x}}_{i_{t}}$ ∈ ℝ^2^ is a coordinate estimation of individual ϑ_*i*_ at time *t*, in a period of observation from 1 to τ_*k*_ for a total of *N* individuals. Using **X** we train a self-organizing map *SOM*_*I*_ containing a set of nodes *S* = {*s*_1_, …, *s*_*m*×*n*_} where *m* is the number of rows and *n* is the number of columns in an hexagonal topology. As a result of the training phase, *SOM*_*I*_ provides a complete topological representation of the environment, where node *s*_*j*_ ∈ *S* represents a mutually exclusive zone in the environment as shown in Figures 3A,B. Representing the environment utilizing a self-organizing map encompasses several advantages including (a) unsupervised learning of the environment's topological configuration, (b) clustering and reduction of data and (c) a simpler way to describe individual's trajectories.

### 3.2. Entity individual

We describe each observed individual ϑ_*i*_ in the environment with an instance of the entity *Individual* hence in a crowd with *N* people detected, a total of *N* instances will be implemented. The hierarchical model of entity *Individual* is presented in Figure 1. We describe the trajectory of ϑ_*i*_ as a discrete-controlled process with a state vector *x*_*i*_*t*__ ∈ ℝ^2^

(2) $$\begin{array}{l} x_{i_{t}}=x_{i_{t-1}}+g_{i_{t}} \end{array}$$

and observation vector *z*_*i*_*t*__ ∈ ℝ^2^

(3) $$\begin{array}{l} z_{i_{t}}=x_{i_{t}}+h_{i_{t}} \end{array}$$

Where *g*_*i*_*k*__ and *h*_*i*_*k*__ represent the process and observation noise, both assumed to be independent, white, with Gaussian distribution. Applying an Extended Kalman Filter (EKF) over the observation and state vectors we obtain an estimation ${\hat{x}}_{i_{t}}$. The trajectory *X*_*i*_ of ϑ_*i*_ is described as a sequence of estimations

(4) $$\begin{array}{l} X_{i}=\left\{{\hat{x}}_{i_{t_{1}}},\ldots ,{\hat{x}}_{i_{t_{k}}};t_{k}\geq 1\right\} \end{array}$$

and ${\left\{X_{k}\right\}}_{k=1}^{N}$ represents the trajectories of individuals ${\left\{\vartheta_{k}\right\}}_{k=1}^{N}$. Using the zones of *S* and *SOM*_*I*_ produced in Section 3.1, we can cluster every estimation ${\hat{x}}_{i_{t}}\in X_{i}$ into a zone *s*_*k*_ ∈ *S* as $SOM_{I}\left({\hat{x}}_{i_{t}}\right)=s_{k}$. Furthermore, we can express the trajectory *X*_*i*_ as a sequence of zones

(5) $$\begin{array}{l} w_{i}=\left\{s_{1},\ldots ,s_{q};s_{j}\in S,q\geq 1\right\} \end{array}$$

where *w*_*i*_ is called a *word*. Words are grouped into a vocabulary *V*_*l*_ = {*w*_1_, …, *w*_*q*_} given the condition that ∀*w*_*a*_, *w*_*b*_ ∈ *V*_*l*_, *s*_1_ ∈ *w*_*a*_ = *s*_1_ ∈ *w*_*b*_ and *s*_*n*_ ∈ *w*_*a*_ = *s*_*m*_ ∈ *w*_*b*_ where |*w*_*a*_| = *n* and |*w*_*b*_| = *m*, that is, words with similar origin and destination. The notion of words provides a simplified way to describe trajectories whereas the notion of vocabularies allows to group trajectories that correspond to the same behavior, aiming to reach the same destination. Hence, each vocabulary *V*_*l*_ indicates a different behavior. Each learned behavior is modeled with two conditional probability distributions (CPDs)

(6) $$\begin{array}{l} \Omega_{s_{\alpha },s_{\beta },s_{b+1},w_{a:b}}^{l}=p\left(s_{\alpha },s_{\beta },s_{b+1}|w_{a:b}\right) \end{array}$$

and

(7) $$\begin{array}{l} \Lambda_{\Delta t,s_{b+1},s_{b}}^{l}=p\left(\Delta t|s_{b+1},s_{b}\right) \end{array}$$

where *s*_α_ is the initial state, *s*_β_ is the final state and *s*_*b*+1_ is the predicted next state given the partial observed trajectory *w*_*a*:*b*_ from time instant *a* to *b* in Equation (6). Δ*t* is the transition time given the current state *s*_*b*_ and the predicted next state *s*_*b*+1_ in Equation (7). The purpose of Equation (6) is to estimate the origin, destination and next state of ϑ_*i*_ by matching *w*_*a*:*b*_ to the most similar existing word *w*_*k*_ with the highest likelihood. On the other hand, Equation (7) estimates the time required for the next transition time. Notice that in both Equations (6) and (7), the superscript *l* is used to indicate the behavior (vocabulary) to which the CPDs correspond to. Given the estimation of trajectory (Equation 6) and transition time (Equation 7) we can proceed to estimate the emotional state using Bayes rule (Equation 8)

(8) $$\begin{array}{r} p\left(E,w_{a:b+1},\Delta t\right)=p\left(E\right)p\left(s_{\alpha },s_{\beta },s_{b+1}|w_{a:b}\right)\\ \;p\left(\Delta t|s_{b+1},s_{b}\right) \end{array}$$

where *E* is the emotional state labeled as positive, normal or negative, and *p*(*E*) is the prior probability learned from the training data and assumed to be uniform. We evaluate Equation (8) for each possible value of *E* in order to find the emotional state with the highest likelihood. Given that the association between behavior and emotion depends on the context of the situation, the rules for labeling are to be determined for each particular scenario. However, in Section 4 we explain the labeling criteria applied to the experiments presented here.

### 3.3. Entity crowd

Supported by the work presented in Le Bon (2007) and Reicher (2012) we argue that a crowd behaves as a collective minded entity and therefore we can model behaviors and infer emotional states for the entity *crowd* in a similar way to that of the entity *individual*. One single instance of the entity *crowd* is employed in a given environment. We start our description of the entity *crowd* by defining a state vector *X*_*C*_*t*__

(9) $$\begin{array}{l} X_{C_{t}}=\left\{x_{1_{t}},\ldots ,x_{N_{t}};x_{i_{t}}\in {\mathbb{R}}^{2}\right\} \end{array}$$

and observation vector *Z*_*C*_*t*__

(10) $$\begin{array}{l} Z_{C_{t}}=\left\{z_{1_{t}},\ldots ,z_{N_{t}};z_{i_{t}}\in {\mathbb{R}}^{2}\right\} \end{array}$$

where *x*_*i*_*t*__ and *z*_*i*_*t*__ are the state and observation vectors of ϑ_*i*_ as defined in Equations (2) and (3), respectively, for a total of *N* individuals. In a similar way we could define ${\hat{X}}_{C_{t}}$ = ${\left\{{\hat{x}}_{i_{t}}\right\}}_{i=1}^{N}$ as an estimation of the state vector of entity *Crowd*, however the difficulty of using that definition is that ${\hat{X}}_{C_{t}}$ is prompt to irregular dimensionality between samples as individuals join or leave the crowd. Instead we define ${\hat{X}}_{C_{t}}$ as

(11) $$\begin{array}{l} {\hat{X}}_{C_{t}}=\left\{{ŷ}_{1_{t}},\ldots ,{ŷ}_{{m\times n}_{t}}\right\} \end{array}$$

where ŷ_*k*_*t*__ is an estimated amount of individuals in zone *s*_*k*_ ∈ *S* at time *t*, for a total of *m* × *n* zones in *S* as produced by *SOM*_*I*_ in Section 3.1. In this sense, the crowd's state vector estimation is implicitly dependent on the estimation of the individuals' trajectories. This definition of ${\hat{X}}_{C_{t}}$ is more advantageous as it provides a vector with uniform dimensionality while maintaining meaningful information. Also, since the focus of ${\hat{X}}_{C_{t}}$ is density estimation rather than trajectory tracking, we could employ crowd density algorithms (Cho et al., 1999; Rahmalan et al., 2006) to achieve this task. We collect the estimations ${\hat{X}}_{C_{t}}$into a training set

(12) $$\begin{array}{l} X_{C}^{T}=\left\{{\hat{X}}_{C_{t_{1}}},\ldots ,{\hat{X}}_{C_{t_{k}}};t_{k}\geq 1\right\} \end{array}$$

and use this set to train a self-organizing map *SOM*_*C*_ that further reduces dimensionality and provides a representation of states transitions. It is important to mention that *SOM*_*C*_ does not provide topological information as *SOM*_*I*_ does. *SOM*_*C*_ is composed by *p* rows, *q* columns and a set of nodes *C* = {*c*_1_, …, *c*_*p*×*q*_} where the node *c*_*k*_ represents a state of the crowd. This enables us to classify each estimation to a state.

For the entity *crowd* we do not define *words* to describe state transition sequences because unlike the entity *individual* where there is a finite trajectory, the sequence of state transitions in a crowd emerges as a cyclic process with people continuously joining and leaving the crowd. As explained in Section 5 we aim to explore the cyclic behaviors of a crowd in a more comprehensive way in future work, but for the work presented here we describe a *crowd* behavior as a first order Markov process with two CPDs to estimate the change of states and transition time

(13) $$\begin{array}{l} {ϒ}_{c_{k},c_{k+1}}=p\left(c_{k+1}|c_{k}\right) \end{array}$$

and

(14) $$\begin{array}{l} \Psi_{\Delta_{t},c_{k}}=p\left(\Delta_{t}|c_{k+1},c_{k}\right) \end{array}$$

where *c*_*k*_ and *c*_*k*+1_ are the current and next state, respectively, and Δ*t* is the transition time. The emotional state of the *crowd* is assigned to be the same as the experienced for the majority of individuals, hence no labeling is applied to specific state or transition time of the entity *crowd*.

## 4. Experiments and results

To validate our proposed model we employ data produced by a realistic crowd simulator first introduced in Chiappino et al. (2012, 2015), based on social forces (Helbing and Molnar, 1995) where each individual in the environment is treated as a particle subject to 2D forces, deriving its motion equations from Newtons law *F* = *ma* and accounting for its motivation as an attraction force pulling the individual toward its destination and repulsive forces from physical objects and other individuals in the environment. We have recreated a scenario similar to that of a train station as shown in Figure 2A, the produced trajectories are plotted in Figure 2B. The information of individual's trajectories is provided directly from the crowd simulator, hence the steps for detection and tracking of people are omitted. Simulations were carried out under different levels of crowdedness, details for the training and testing datasets produced from simulations are presented in Table 1.

**Table 1:** **Parameters of training and testing datasets produced from simulations**.

**Parameter**	**Training dataset**	**Test dataset**
Duration of dataset	5 h	5 h
Number of positive trajectories	978	894
Number of normal trajectories	1770	1815
Number of negative trajectories	252	291
Total number of trajectories	3000	3000

The self-organizing maps *SOM*_*I*_ and *SOM*_*C*_ are initialized with similar parameters. The set of neurons on each *SOM* is initialized with random weights and in a hexagonal arrangement spread across the corresponding input space. Distance between neurons is calculated by the number of links among them. The initial neighborhood size is 3 with 100 steps for the ordering phase. The training phase is done over 500 epochs by competitive layer but without bias, updating the winning neuron and all other neurons within the given neighborhood using Kohonen rule.

The first task addressed is to use the trajectory of individuals to obtain a topological representation of the environment with the help of a self-organizing map *SOM*_*I*_ as shown in Figures 3A,B. We can observe from Figure 3C the distribution of training data among zones after the clustering process, which is important when describing trajectories, larger zones indicate that more trajectories traverse this area whereas the opposite is also true for smaller zones. The decision of how many zones to employ to describe trajectories has a direct impact on the reliability of our model to estimate the emotion of individuals; this happens because we describe the behavior of individuals by transition of zones, and with fewer zones there is a higher uncertainty of the motion of individuals. In these experiments, *SOM*_*I*_ is composed of 100 zones (10 rows and 10 columns) in a hexagonal topology. After testing our model with different dimensions, we found this size to be a suitable balance between predictability and topological representativeness.

**Figure 4:**
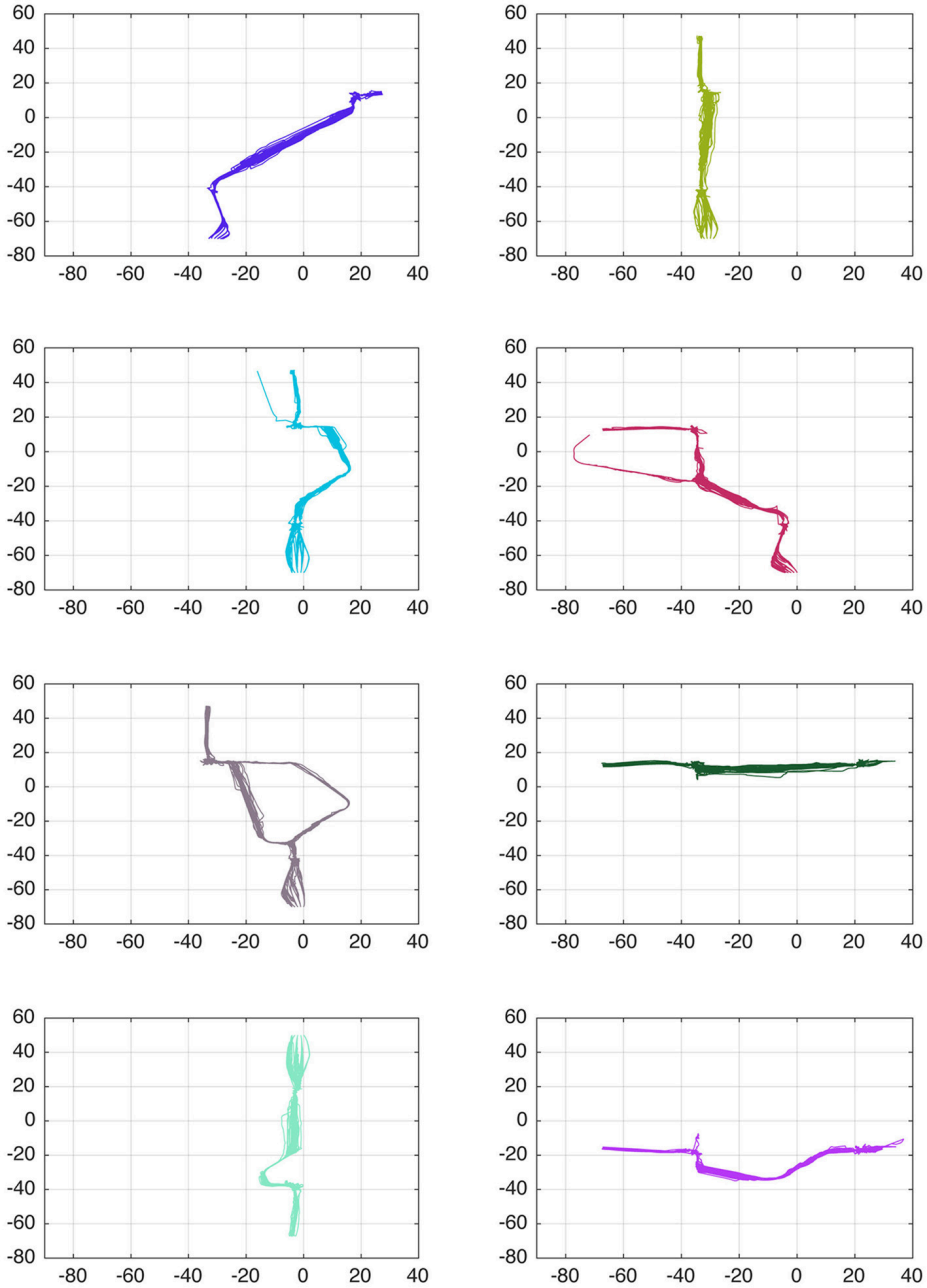
**Examples of learned behaviors from the trajectories in the training phase, a total of 41 different behaviors where identified**. Colors are assigned randomly.

Employing *SOM*_*I*_, the trajectories of the training set were evaluated, and a total of 41 different behaviors were identified, a few examples of the learned behaviors are shown in Figure 4.

**Figure 5:**
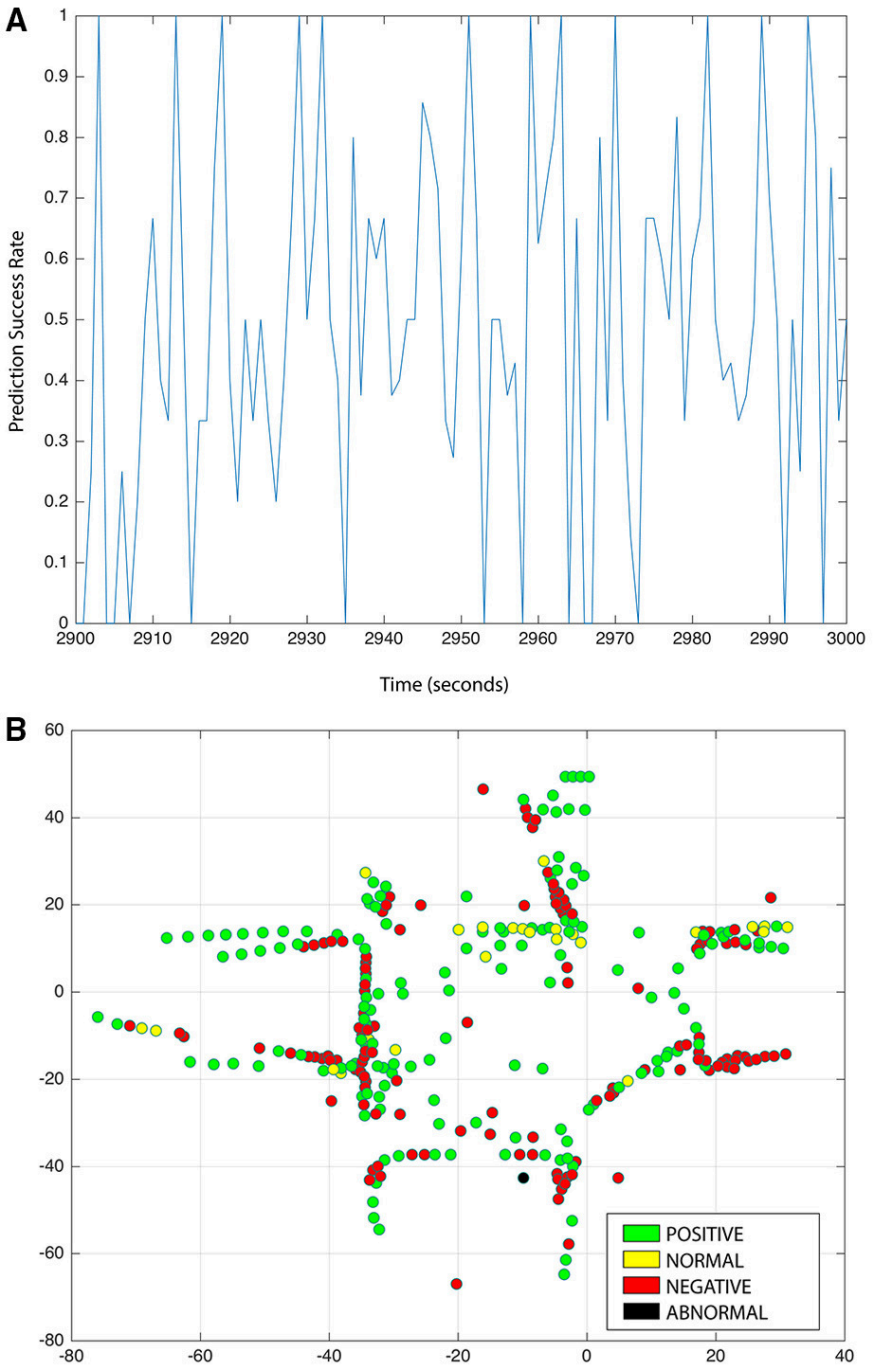
**(A)** Overall success rate in behavior prediction of individuals. **(B)** Online emotion estimation of individuals.

The scenario replicated in the experiments corresponds to that of a train station. Hence, the criteria for labeling behaviors follows from the assumption that people aim to reach their destination in the briefest possible time. The behaviors with the minimum number of state transitions and the shortest transition time are labeled with a positive emotion. The behaviors with the higher frequency of occurrence are associated with a normal emotion. Finally, the behaviors with the highest number of transitions and longer transition time are assigned a negative emotion. During the testing phase, for the purpose of estimating emotional state on individuals, our hierarchical model predicts the zones transitions and transition time for each individual in real time based on the learned behaviors. In our model, the accuracy level to estimate the emotion of individuals depends on the model's capability to predict the individual's behavior. In Figure 5A we show the behavior prediction success rate during a period of 100 s where the average rate was of 76%. Throughout the entire length of the simulations, the prediction success rate oscillated between 74 and 82%. A summary of the model's performance to estimate emotional states is presented in Table 2. In Figure 5B we present a snapshot of the online emotion estimation of individuals.

**Table 2:** **Confusion matrix of emotional state estimation based on individuals' behavior**.

**Actual**	**Estimation**		
	**Positive (%)**	**Normal (%)**	**Negative (%)**
Positive	71	23	6
Normal	14	83	3
Negative	4	21	75

**Figure 6:**
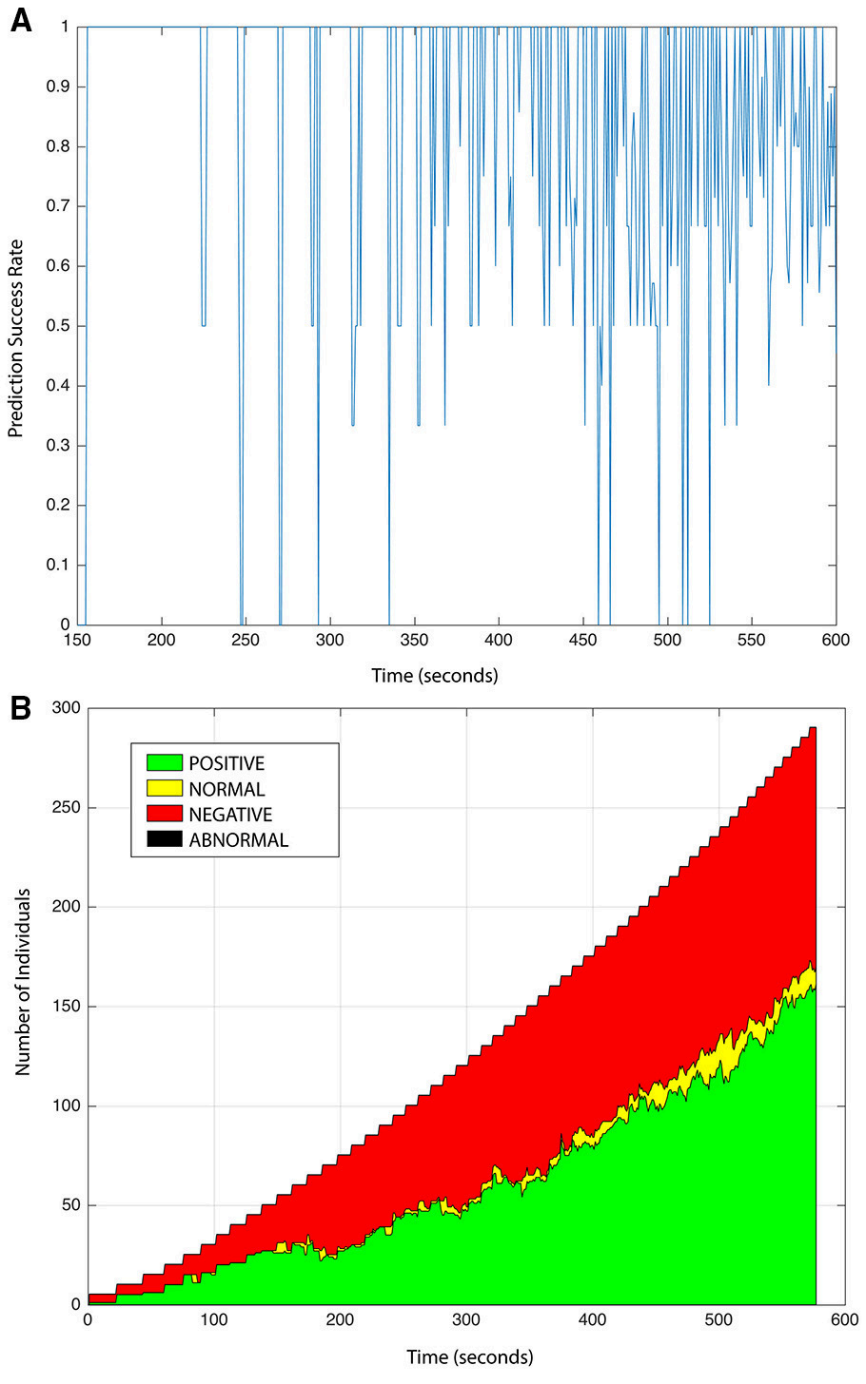
**(A)** Success rate in behavior prediction of *crowd* entity. **(B)** Online emotion estimation of the crowd.

The behavior of the *crowd* is described with a second self-organizing map *SOM*_*C*_, also composed of 100 zones (10 rows and 10 columns), which allow us to build a probabilistic model for its behavior. However, unlike *SOM*_*I*_, a plot of *SOM*_*C*_ does not provide a visual semantic due to the high dimensionality of its state vector. Using the same simulations applied to test our model for individuals, we test the ability of the crowd model to predict its behavior, that is, the next state and transition time among states of *SOM*_*C*_. In Figure 6A we can observe the behavior prediction success rate of the *crowd* oscillating more consistently between 50 and 94%, with an average rate of 81%. In the work proposed here, the emotional state of the *crowd* is not correlated to its behavior, instead is assigned to be the same as the experienced for the majority of individuals. In Figure 6B we display the summary of detected emotions in an observation period of 600 s, during which a positive emotion becomes predominant as the number of individuals joining the crowd increases.

## 5. Conclusions and future work

In this work, we presented a robust model for the estimation of emotions on single individuals and the crowd as a whole, under complex crowded environments. In comparison with (Baig et al., 2014), our approach provides significant improvements: (1) accounts for scenarios with multiple origin and destination points, (2) introduces the idea of vocabularies to describe behaviors which help to reduce data sparsity, and (3) explores the idea of the crowd as a single entity with its own behavior and emotional state.

Our overall hypothesis is that crowd emotion is a combination of individual's emotion estimation, as suggested by neuroscience studies (de Gelder et al., 2010). In this particular study, we have a rather simple model that treats the crowd emotion as a sum of the emotions of the individuals in the crowd. The emotion of the crowd is estimated by a deviation of normal patterns and speed of movement identified in normal situations.

The approach presented here is applicable to real-life crowded environments for monitoring automation intended to identify and prevent dangerous situations as well as to improve crowd control. Furthermore, contributions of this nature are essential for the development of robust cognitive dynamic systems intended for smart cities.

Future development of this work will focus on extending the model to consider the interaction of individual and crowd emotions enabling us to explore causality and contagion of emotional states among individuals and its impact in the crowd as a whole. The result of the simulations performed show the behavior of the crowd to emerge in a cyclic manner; we are interested in further explore this phenomenon and to provide a more comprehensive model for describing such behavior of the crowd. Finally, we aim to extend this model to enable its use in first person perspective models and applications.

## Author contributions

The central idea of this work was developed jointly with CR throughout extensive discussions. MB implemented modifications to the employed software to suit the experiments needs. LM helped in several technical aspects to facilitate the execution of the simulations. EB provided essential insights in the fundamentals of emotion theory and contributed to writing the manuscript. MR assisted with valuable insights and revision of the manuscript. All authors contributed significantly to the work presented in this paper.

## Funding

This work was produced under the program of Erasmus Mundus Joint Doctorate in Interactive and Cognitive Environments (EMJD ICE), funded by the Education, Audiovisual and Culture Executive Agency (EACEA).

### Conflict of interest statement

The authors declare that the research was conducted in the absence of any commercial or financial relationships that could be construed as a potential conflict of interest.
